# Selected Papers in 2023–2024 in the “Polymer Membranes and Films” Section

**DOI:** 10.3390/polym17233217

**Published:** 2025-12-03

**Authors:** Yuekun Lai

**Affiliations:** 1 School of Chemical Engineering, Fuzhou University, Fuzhou 350108, China; yklai@fzu.edu.cn; 2 State Key Laboratory of New Textile Materials and Advanced Processing, Wuhan Textile University, Wuhan 430073, China

In recent years, research within the “Polymer Membranes and Films” Section has increasingly reflected a convergence of molecular engineering, interfacial design, and processing innovation aimed at achieving both performance enhancement and environmental responsibility. The selected papers illustrate this trajectory across diverse material platforms, ranging from functional membranes and bio-based composites to surface-engineering coatings and supramolecular solvent systems. Together, they highlight how structural control—from nanoscale assemblies to macroscale film architectures—can be strategically leveraged to tune transport behavior, durability, stability, and multifunctionality. This editorial provides integrated overview and identifies overarching scientific themes and emerging methodological needs, including the refinement of structure–property relationships, improved scalability of fabrication techniques, and the incorporation of sustainability and life-cycle criteria into next-generation polymer technologies.

“Coating Materials to Increase the Stability of Liposomes” [1] reviews the design principles and materials strategies used to enhance the physical and chemical stability of liposomes for drug, nutraceutical, and food applications. The article outlines how fragile phospholipid bilayers suffer from thermal degradation, light-induced oxidation, pH variations, and enzymatic breakdown, and it organizes how coating materials—from saccharides and polysaccharides to proteins, polymers, and hybrid multilayers—modulate membrane rigidity, permeability, and gastrointestinal resistance. Drawing on comparisons across chitosan, alginate, pectin, whey protein, polyethylene glycol (PEG), and other functional biopolymers, the review shows how surface engineering extends circulation time, improves encapsulation efficiency, and enables controlled or site-specific release. It further highlights how polymer–lipid interactions, electrostatic layer-by-layer assembly, and structural modifications (e.g., PEG alternatives and chitosan derivatives) allow fine-tuning of release behavior and biological interactions, while identifying challenges such as coating uniformity and cytotoxicity. Recent directions include multi-component shell design for enhanced gastric survival, natural macromolecule coatings for PEG-free stabilization, and applications in both pharmaceuticals and functional foods. Overall, the review positions coated liposomes as a versatile platform for next-generation delivery systems and outlines key considerations for translating coating strategies into industrial products.

“The Difference in Performance and Compatibility between Crystalline and Amorphous Filler in Mixed Matrix Membranes for Gas Separation (MMMs)” [2] examines how filler morphology governs gas-transport behavior, interfacial stability, and long-term performance in polymer–filler hybrid membranes. The review contrasts crystalline fillers such as metal–organic frameworks (MOFs), zeolites, and covalent organic frameworks (COFs)—with their well-defined porosity and molecular-sieving selectivity—against amorphous fillers including porous organic polymers (POPs), porous aromatic frameworks (PAFs), and polymers of intrinsic microporosity (PIMs) that tend to offer better interfacial compatibility and reduced microvoid formation. It analyzes how permeability–selectivity trade-offs are modulated through filler porosity, functionalization, particle dispersion, and polymer–filler interactions, and discusses the consequences of physical aging in high-free-volume matrices. Transport phenomena are contextualized using Maxwell-type effective-medium theories, resistance-based models, and dual-mode sorption descriptions, highlighting deviations caused by interfacial defects or synergistic diffusion pathways. In addition, the review outlines practical strategies—such as post-synthetic modification of MOFs, incorporation of ionic liquids, or the use of ternary compatibilizing components—to overcome polymer–filler adhesion bottlenecks and stabilize performance under industrial conditions. Overall, the article provides a framework for aligning filler chemistry, morphology, and processing routes to accelerate the translation of MMMs from laboratory demonstrations to durable, scalable gas-separation technologies.

“Thermal Behavior of Poly(vinyl alcohol) in the Form of Physically Crosslinked Film” [3] investigates the distinct thermal properties of poly(vinyl alcohol) (PVA) films cast from H_2_O and D_2_O solutions compared to raw powder. The study demonstrates that solution casting induces physical crosslinking through a macromolecular hydrogen-bonding network, which enhances thermal stability and allows the film to swell as a hydrogel while retaining its shape. Unlike the raw powder, which exhibits rapid mass loss due to volatile impurities like methyl acetate, the crosslinked film displays a delayed and slower decomposition rate. Advanced characterization using differential scanning calorimetry (DSC), thermogravimetric analysis (TGA), and Fourier transform infrared spectroscopy (FTIR) reveals that the material’s thermal events—traditionally identified as glass transition (75–85 °C) and melting (~200 °C)—are actually “thermochemical transitions” where softening occurs simultaneously with partial decomposition. Furthermore, isotopic exchange with D_2_O indicates that strongly bound water molecules act as integral crosslinkers within the polymer matrix. Overall, the research redefines the understanding of PVA’s thermal behavior by highlighting the governing influence of physical crosslinking and impurity removal on stability and transition mechanisms.

“Formation of Porous Structures and Crystalline Phases in Poly(vinylidene fluoride) Membranes Prepared with Nonsolvent-Induced Phase Separation—Roles of Solvent Polarity” [4] reports the impact of solvent choice—specifically HMPA, NMP, DMAc, and TEP—on the morphology and crystallinity of PVDF membranes fabricated via nonsolvent-induced phase separation (NIPS). The study establishes that both the water permeability and the proportion of polar crystalline phases (specifically the β-phase) increase monotonously with the solvent’s dipole moment. Through in situ FTIR analysis, the researchers demonstrated that solvents with higher dipole moments produce more viscous casting solutions, which significantly shows the solvent removal rate during coagulation. This delayed removal creates prolonged “solvent-induced” polar crystals and highly porous structures. In contrast, solvents with lower dipole moments or faster removal rates lead to “solvent-poor” conditions that favor the kinetically stable, non-polar α-phase and result in denser membranes with reduced permeability.

“Review of Synthesis and Separation Application of Metal–Organic Framework-Based Mixed-Matrix Membranes” [5] outlines the fabrication strategies for Metal–Organic Framework-based (MOF-based) membranes, categorizing techniques into in situ growth, secondary growth, and electrochemical deposition while evaluating their respective advantages in controlling crystal orientation and density. It details how specific fillers like Zeolite Imidazolate Frameworks (ZIF), University of Oslo (UIO), and Materials of Institute Lavoisier (MIL) are integrated into polymer matrices to overcome the trade-off between permeability and selectivity, highlighting modification strategies like ionic liquids and core–shell structures to enhance compatibility. The article compiles performance data across diverse separation systems, illustrating how MOF porosity and functionalization drive efficiency in lithium–sulfur battery separators by inhibiting polysulfide shuttling, as well as in seawater desalination and heavy metal removal from wastewater. The discussion extends to liquid–liquid and gas-phase applications, demonstrating the efficacy of superwetting membranes for oil–water separation and the high affinity of amine-modified frameworks for carbon dioxide capture and particulate matter filtration. Finally, practical challenges regarding high production costs and large-scale manufacturing are identified, emphasizing the need for low-cost ligands and industrial adaptation to realize the full potential of these advanced separation materials.

“Advancements in the Application of Sulfonated Poly(Ether Ether Ketone) (SPEEK) and Its Organic Composite Membranes for Proton Exchange Membrane Fuel Cells (PEMFCs)” [6] reviews recent application progress of sulfonated SPEEK and its organic composite membranes in proton exchange membrane fuel cells (PEMFCs). It summarizes how sulfonation degree, ion-exchange capacity, and swelling behavior govern proton transport, dimensional stability, and oxidative resistance, and shows that property tuning is achievable by adjusting molecular architectures and processing conditions. The article focuses on summarizing the design strategies of SPEEK composite membranes, especially by introducing inorganic fillers (such as, SiO_2_, TiO_2_, and zeolites) or high-molecular polymers (such as PVDF and PBI) to construct organic composite membranes, thereby solving the problems of low proton conductivity and high methanol permeability of pure SPEEK membranes in high-temperature and low-humidity environments. Performance data across PEMFCs and direct methanol fuel cells (DMFCs) are compiled, highlighting the synergistic effect of SPEEK/PVDF nanofiber composite membranes in maintaining high mechanical stability and low methanol permeability. The review also extends the discussion to long-term durability, chemical stability, and fuel-cell operating challenges, identifying bottlenecks in commercial deployment and outlining directions for next-generation functional fillers and structural designs that could enable high-performance, application-ready proton-exchange membranes.

“Transparent Superhydrophobic and Self-Cleaning Coating” [7] surveys a simple spray-coating strategy to fabricate highly transparent superhydrophobic films using hydrophobic fumed silica (HF-SiO_2_) and waterborne polyurethane (WPU) as a binary composite system. The authors disperse HF-SiO_2_ and WPU in ethanol to form a uniform suspension and optimize deposition by controlling spray pressure, distance, and curing to obtain a ~19.6 μm coating with well-distributed micro-/nanostructures. Scanning electron microscopy (SEM), laser scanning confocal microscopy (LSCM), energy-dispersive X-ray spectroscopy (EDS), FTIR, and X-ray photoelectron spectroscopy (XPS) collectively confirm a hierarchical rough morphology composed of micron-scale protrusions and a nanoscale porous network, along with characteristic Si-O-Si, Si-C, and C-O bonding, which together provide both roughness and reduced surface energy. Surface wettability tests reveal a water contact angle of 158.7 ± 1.5° and a sliding angle of 6.2 ± 1.8°, while high-speed imaging (solid–liquid contact time ~14 ms) demonstrates rapid droplet rebound consistent with Cassie–Baxter wetting and ultra-low adhesion. Despite the hierarchical roughness, ultraviolet-visible spectroscopy (UV-Vis) measurement shows that the coating maintains 83.4% transmittance at 500 nm—96.1% of bare glass—attributed to its uniform nanoporous structure and limited thickness. The coating also provides robust self-cleaning behavior: contaminants including sand, potassium chromate, CuCl_2_·2H_2_O_2_, and SiC are readily removed by rolling droplets, mimicking lotus-leaf behavior. By integrating high transparency with stable superhydrophobicity, the HF-SiO_2_@WPU system overcomes the typical trade-off between roughness and optical clarity. The study highlights a scalable, low-cost, fluorine-free route for transparent protective coatings with promising applicability in optical windows, architectural glass, solar panels, and other devices requiring low adhesion and self-cleaning surfaces.

“Characterization of Polyvinyl Alcohol (PVA)/Polyacrylic Acid (PAA) Composite Film-Forming Solutions and Resulting Films as Affected by Beeswax Content” [8] presents a physically–chemically crosslinked hydrogel synthesized by combining freeze-thawed PVA with thermally polymerized PAA to achieve a mechanically reinforced and highly swollen network. The authors confirm polymer blending and network formation through FTIR, where broad -OH stretching and characteristic carbonyl absorptions indicate hydrogen bonding between PVA chains and the PAA backbone. Thermal behavior assessed via DSC reveals the loss of crystallinity in PVA after PAA incorporation, consistent with chain disruption and enhanced amorphous character. SEM imaging shows a heterogeneous porous morphology, with larger pore domains than neat PVA, supporting the material’s improved water uptake. Swelling tests further demonstrate dramatically higher equilibrium swelling ratios in PVA/PAA compared to pure PVA, attributed to the ionizable carboxyl groups and the osmotic driving force between polymer and medium. Mechanical evaluation shows that despite reduced crystallinity, the hybrid hydrogel maintains sufficient structural integrity due to the dual-network effect: PVA provides physical crosslinks, while PAA contributes chemical anchoring. Together, these analyses show that blending PVA with PAA produces a hydrogel with tunable swelling, modified thermal transitions, and enhanced network heterogeneity, offering a versatile platform for biomedical, environmental, and soft-material applications.

“A Review on the Application of Deep Eutectic Solvents in Polymer-Based Membrane Preparation for Environmental Separation Technologies” [9] surveys the formation, properties, and application pathways of deep eutectic solvents (DESs), focusing on how hydrogen-bond donor/acceptor pairings generate eutectic mixtures with low volatility, high thermal stability, and tunable polarity and viscosity for extraction, catalysis, electrochemistry, and materials processing. The review organizes DES performance around physicochemical descriptors—hydrogen-bonding strength, ionicity, viscosity, conductivity, and water sensitivity—and shows how these parameters determine mass-transfer efficiency in metal recovery, selectivity in biomass fractionation, and electrode/electrolyte interfacial behavior in electrochemical systems. It compiles comparative data demonstrating that DESs can outperform conventional organic solvents in metal ion separation, dye removal, and carbohydrate or lignin dissolution, while maintaining low toxicity and biodegradability. Blending strategies, hydration control, and component selection are presented as levers to adjust solvating ability and kinetics. The article contrasts choline-chloride-based DESs with type-III natural DESs, highlighting greener synthesis routes and reduced environmental impact. Practical limitations are also identified, including high viscosity that restricts diffusion, incomplete understanding of microstructure and solvation mechanisms, and scale-up challenges arising from cost, recyclability, and process integration. These considerations lead to a roadmap emphasizing mechanistic modeling, viscosity-lowering strategies, and standardized stability/toxicity testing to accelerate DES adoption in sustainable chemical processes.

Finally, “Surface Modification of Flax Fibers with TMCTS-Based PECVD for Improved Thermo-Mechanical Properties of PLA-Flax Fiber Composites” [10] monitors a plasma-enhanced chemical vapor deposition (PECVD) route that deposits hydrophobic organosilicon-like thin films onto flax fibers using tetramethylcyclotetrasiloxane (TMCTS) as the precursor. The study organizes fiber modification around plasma pretreatment, TMCTS plasma polymerization, and its influence on interfacial chemistry and mechanical reinforcement within PLA-based composites. FTIR, XPS, and SEM analyses demonstrate wax removal, the emergence of carbonyl functionalities after N_2_ plasma activation, and the formation of uniform Si-O-Si-rich coatings that reshape surface polarity and chemical composition. These changes enhance fiber–matrix compatibility, enabling stronger load transfer and reduced moisture sensitivity. Dynamic mechanical testing shows substantial increases in storage modulus and loss modulus in TMCTS-treated composites, indicating improved stiffness and damping relative to untreated fibers. Together, the work highlights how PECVD-derived organosilicon layers provide a controllable, solvent-free pathway to tailor natural fiber surface, mitigate interfacial defects, and deliver PLA/flax composites with superior mechanical integrity and thermal–mechanical stability, supporting broader adoption of sustainable bio-composites.

The ten contributions highlighted demonstrate how the evolving synergy between polymer chemistry, interface engineering, and advanced processing can simultaneously advance functionality, sustainability, and application readiness. From bio-inspired surface coatings to energy-relevant membranes, bio-based composites, and solvent-innovative fabrication routes, these studies collectively reinforce the momentum toward scalable, durable, and environmentally compatible polymer technologies. I hope these examples stimulate deeper dialogue and collaboration across academic and industrial communities, further accelerating the development of standardized testing frameworks, predictive design tools, and manufacturing strategies. I also look forward to the continued growth of the “Polymer Membranes and Films” Section as an influential platform for rigorous, forward-looking research.
